# “I have to fight for them to investigate things”: a qualitative exploration of physical and mental healthcare for women diagnosed with mental illness

**DOI:** 10.3389/fpubh.2024.1360561

**Published:** 2024-04-30

**Authors:** Tessa Zirnsak, Rosiel Elwyn, Grace McLoughlan, Esther Le Couteur, Chloe Green, Nicholas Hill, Russell Roberts, Chris Maylea

**Affiliations:** ^1^Social Work and Social Policy, Department Clinical and Community Health, La Trobe University, Bundoora, VIC, Australia; ^2^Neuroscience and Psychiatry, Thompson Institute, University of the Sunshine Coast, Birtinya, QLD, Australia; ^3^School of Law, La Trobe University, Melbourne, VIC, Australia; ^4^School of Global, Urban and Social Studies, RMIT University, Melbourne, VIC, Australia; ^5^Department of English, Drama and Film, University College Dublin, Dublin, Ireland; ^6^School of Social and Political Sciences, University of Melbourne, Melbourne, VIC, Australia; ^7^School of Business, Charles Sturt University, Bathurst, NSW, Australia

**Keywords:** mental illness, women, physical health, patient-doctor relationship, self-determination, diagnosis

## Abstract

**Introduction:**

Women play a significant role in the management of their own healthcare and that of others, however women diagnosed with mental illness and physical health concerns experience significant health inequalities as compared to people living without mental health concerns.

**Methods:**

In this paper, we reflect on the experiences of 20 cis women diagnosed with mental and physical health concerns who agreed to be a part of this research. This qualitative study is part of the larger Healthtalk Australia research project which was not gender specific. Female participants shared many experiences of mental and physical healthcare in interviews with researchers that pointed to the need for a gendered approach to addressing health inequalities. Consequently, we iteratively consolidated transcripts of interviews with participants into thematic categories facilitated by NVIVO 12.

**Results:**

We identified two broad themes and a set of subthemes: in the doctor’s office – experience of labelling; negotiating medications; and interactions with physical and mental health, and outside the doctor’s office – responses to trauma, financial concerns, and reliance on participant’s internal resources to get healthcare needs met.

**Discussion:**

We conclude that participants in this study undertook significant work to manage their own healthcare needs, despite being challenged by clinicians and systems that failed to see them as whole people with expertise regarding their own health.

## Introduction

People diagnosed with mental illness die younger and more frequently from preventable causes than the general population (1–3). A recent study suggested that only 1 in 5 general practitioners asked about the physical health of clients diagnosed with mental illness (4). People diagnosed with mental illness are more likely to experience diagnostic overshadowing, whereby clinicians – which we defined as general practitioners, medical specialists, mental health workers (excluding peer workers) and allied health practitioners – wrongly attribute physical health symptoms to mental health diagnoses (5). In one study by Nash (6), people diagnosed with mental illness reported being told by their doctors that they were imagining physical health symptoms for which they were seeking treatment [see also (7, 8)]. In a review of the literature on the experiences of people diagnosed with mental illness seeing physical healthcare, our team identified that healthcare is a human right, and yet, health systems and professionals fail to ensure this right is actualised (9).

The physical health experiences of women who access mental health services are under researched and under-valued (9). Women are statistically more likely to experience mental and physical health disablement because their needs as they understand them are misconstrued by practitioners and experiences often psychopathologised (10–12). The diagnostic labels received by women can be incongruent with their experiences and disempowering (13). This is likely to be at least partly because of how social categories of illness and women are constructed; Barr et al. (10) illustrate how mental and physical healthcare provision constructed through policy, funding, workforce, and research and data collection reproduce and reinforcing gender disparities rather than improve mental and physical health outcomes.

In the present study, we spoke to women who were diagnosed with mental illness and were also experiencing at least one physical health concern. We found that women’s experiences of mental and physical health systems had wide ranging affects which impacted their identity, shaped interactions with health professionals and influenced social relationships. In this paper, we share a small part of their stories in the hope of improving our collective understanding of the experiences of women diagnosed with mental illness and living with physical health concerns.

We adopted an inductive approach to the analysis of the interview data which allows women to speak for themselves, particularly how they interpreted all diagnoses they had received. Consequently, this paper addresses women’s experiences accessing physical healthcare, mental healthcare, accessing both healthcare systems, as well as the impact of diagnosis and illness on their identities and how they navigate the impact of their health needs on their lives. While there is ample literature on women’s health as related to childbearing, there is a need to go beyond seeing women’s unique needs as only related to reproductive capacity (14), and instead focus on what women themselves say is important. This paper addresses this gap in the literature. The findings of this paper support the idea that women’s access to and experiences of mental and physical healthcare must be improved. Removing barriers to healthcare for women, as well as improving understanding of gender disparities within healthcare and improving outcomes is a social justice issue related to cultural ideas of womanhood, illness, and mental illness.

## Existing evidence

Women commonly experience epistemic injustice in their healthcare experiences, leading to structural barriers in accessing quality healthcare, and widening existing health disparities (15–17). Women are more likely to experience physical health problems and receive diagnoses of mental disorders (18). These challenges and the existing literature on them are worth mentioning to foreground the context in which participants shared their experiences with the research team.

Health and wellbeing are influenced by genetic, neurobiological, environmental, and social factors. The social category of womanhood is closely linked to sociocultural and institutional milieus that may impact engagement in health services in unique ways for women (19, 20). These include adverse experiences such as discrimination and gender inequality which can both lead women to delay access healthcare and diminish the quality of care they receive [(15, 21, 22); (23)]. For example, women are also more likely to face vulnerability and exposure to violence, including family and sexual violence (10, 24), which they may need health services to navigate. Inadequate recognition of women’s social roles and individual health experiences produced gendered has increased gender-based health inequalities, including increased occurrence of preventable morbidity and mortality (25).

Health inequities experienced by women in their communities and relationships are compounded by inequities in the health system, such as medical gaslighting (15), also known as health-related communicative disenfranchisement (26–29). Medical gaslighting refers to the denial and dismissal of symptoms, invalidation and disregard of patient concerns and wishes, refusal of screening, referral and treatment, gender bias in healthcare, stigmatisation of mental ill health by healthcare professionals, and inadequate care experienced by individuals in medical settings (15–17, 19, 30–35). For example, women may experience being dismissed as lacking credibility when reporting illness symptoms (16, 17, 26, 31, 35).

A systematic review of the psychological impact of medical gaslighting on women (15) found the healthcare experiences of women to be overwhelmingly negative and encompassing medical gaslighting, leading to delayed diagnosis, the need for self-advocacy, worsening health conditions and trauma (15). Experiencing medical gaslighting can lead to women suppressing emotion in clinical settings out of fear of being stigmatised and dismissed (35), using comprehensive self-management and communication strategies, and self-advocacy in an effort to get needs met and avoid further inequity (15, 34–36), feeling that symptoms must need to be severe in order to warrant seeking medical care (36), and feelings of grief and loss from years spent undiagnosed and untreated (37, 38). Due to medical gaslighting, women may also distrust, fear and avoid health services (17, 32, 34, 39), and under-report their symptoms (40). This can increase vulnerability to illness, for example via delayed diagnosis (16, 38), and worsening symptoms (15, 17).

Women may experience multiple, intersecting discrimination that increase risk of medical gaslighting (i.e., sexism, racism, ableism, ageism, sizeism) (16, 19, 32, 34). For example, their gender and age leading to perceived lack of credibility, dismissal, and misdiagnosis (32, 34), or women’s intersections of gender, race, or weight may lead to stigmatising health experiences based on these characteristics (19, 34). Co-diagnosis of a mental and physical health condition can increase the likelihood that women will be stigmatised and dismissed as imagining or fabricating somatic symptoms (32).

These gendered inequalities in healthcare outcomes have historical precedent. Professor of Psychology Jane Ussher (41) has claimed that ‘women outnumber men in diagnoses of madness, from the “hysteria” of the eighteenth and nineteenth centuries, to “neurotic” and mood disorders in the twentieth and twenty-first’. Gendered healthcare interventions of the twentieth and twenty-first centuries have been related to eugenic ideas of womanhood. Until the 1980’s in the United States, women placed in residential care were sterilised (42). In 2007, (43) reported that women accessing treatment in residential settings in the United Kingdom having their menstrual periods tracked by staff without their input or consent.

Today, women are still more likely to be diagnosed with mental illness – and some commentators note that this is due to shifting understandings of what it means to experience mental distress (44). Critiques of psychiatry demonstrate how cisgender women’s experiences of socially produced distress are often repackaged as psychiatric diagnoses (45). The lack of attention to social and cultural contexts that women live in means mental distress is wrongly understood as an individual condition requiring biomedical intervention. In an empirical study by Lafrance (46), participants felt the medicalisation of distress provided some validation of their suffering but delegitimated their social experience of distress.

## Methods

The data analysed in this paper is from a qualitative study which included leadership from academics and community members diagnosed with mental illness and physical health concerns. The purpose of the broader study was to investigate the physical health experiences of people who access mental health services. However, women participants expressed perspectives related to gender, prompting further interrogation into their experiences. Human Research Ethics approval was granted by the RMIT University Human Research Ethics Committee (Project #23687).

The project’s Consumer Leadership Advisory Group (CLEAV) included a range of people with experiences of accessing services for physical and mental health. Members were recruited as experts on engaging with physical and mental health services, with the understanding that they would provide feedback from this lived experience perspective. They met across the course of the project and provided feedback on all aspects of the project plan, ethics, data collection, analysis, and design of the online resource. Wherever possible, project decisions were taken to the CLEAV for discussion and feedback. This group was not engaged in specifically addressing the experiences of women, but gender-specific comments were made by members of this group.

This research took place during the COVID-19 pandemic. Australian jurisdictions were subjected to stay-at-home orders and where possible, community-based healthcare provisions were moved online (47). While some participants did mention COVID-19, the impact of COVID-19 did not emerge as an overall theme in the relevant data. The effects of the pandemic may have been irrelevant because women’s accounts of mental health diagnoses and physical health problems often predated the pandemic.

### Participants

The 20 women whose stories informed this project came from diverse socioeconomic and cultural backgrounds. Participants were aged between 21 and 66 years old at the time of their interviews. They had lived in various countries but all lived in Australia at the time of the interviews. Participants’ educational backgrounds varied, ranging from having left secondary school without completing Year 12 through to the completion of masters degrees. This paper only addresses the experiences of cisgender women. No participants within the larger study identified as transgender or non-binary, however, researchers relied on and trusted participants reporting of their gender without question, and this basic respect would have been extended to gender diverse and gender non-conforming people if any chose to participate.

All participants had received at least one mental health diagnosis and physical health condition. Based on advice from CLEAV, we did not ask participants to list their diagnoses, instead asking participants only if they had received a diagnosis of both mental and physical health conditions and allowing them to opt in if they judged this as true for them. This advice was given on the basis that diagnosis was often disempowering, positioning people as “ill” based on external criteria rather than on the person’s own experience and strategies for navigating unsympathetic health systems and past trauma. Further, the CLEAV did not want participants in this study to be defined by researchers based on their diagnosis. Some participants did opt to share diagnoses in their interview, but this was not requested.

In-depth interviews were conducted from March to December 2021. A copy of the interview schedule is available as Supplementary File 1. Participants engaged in a single interview with one or two members of the research team, including one researcher with lived experience as a woman diagnosed with mental illness and physical health concerns. Interviews were conducted online or face to face, and were either video or audio recorded, according to participant preference. Recordings were professionally transcribed and NVIVO was used for data management and analysis.

A narrative approach to the interviews was adopted which allowed participants to tell their story as they understood it (48). Interviewers typically started the interviews by asking the participant “could you tell me about your experience of your physical health and the care and treatment you have accessed since your first experience of a mental health issue?.” Participants could then respond as they saw fit, and the interviewer would ask questions based on what the participant shared, or ask follow-up questions from the interview schedule, such as “what healthcare services are you accessing?,” “what are your current health concerns and your approach to managing these?,” and “what is the impact of your mental health on your physical health?”

### Data analysis

Four researchers worked to code the transcripts, and each transcript was reviewed by two members of this sub-team (RE, GM, CG, CM). Completed coding was passed on to TZ who used inductive coding following Braun and Clarke (49) to identify eight key themes across two domains: inside the doctor’s office and outside of it. TZ drafted a version of the analysis based on these two domains and circulated it to all authors who provided feedback on the validity of the themes based on their own understandings of the data.

## Findings

Participants described many challenges they experienced when trying to get their physical and mental health needs met. Many of these challenges impacted their everyday lives. We present our analysis across two overarching themes and several sub-themes:

- In the doctor’s office: women experienced labelling, negotiated medications and side effects, and discussed interactions with physical and mental health experiences.
- Outside the doctor’s office: women were navigating responses to trauma, managing financial concerns, and relying on their own internal resources to get their healthcare needs met. These themes are explored below.

### In the doctor’s office

The women in this study came to medical environments with their own experiences, perspectives, and values. Often these were deeply shaped by their experiences of gender but also interactions with mental health, medical, and other allied health professionals. Participants felt that clinicians came to their appointments with their own predefined understandings of who they were and what they needed.

#### Labelling

Participants experienced labelling as designations and assumptions that clinicians assigned to them that had negative consequences for their sense of self. Many had complex relationships with the label they had received, based on feeling pathologised or dismissed. One participant expressed that they would like more say over how they are viewed by clinicians:

‘Oh well, I can think of practical things like, you know, well look at me as a mother, what does a mother need, or do not look at me as a diagnosis, do not look at me as a risk.’ – Participant 13.

This participant resists how psychiatric diagnoses and perceptions of ‘risk’ erased her experience as mother to a set of symptoms. The label of mental illness deeply shaped women’s attempt to access mental and physical healthcare. Another participant felt they were misdiagnosed with an anxiety disorder, which impacted the willingness of clinicians to treat her physical symptoms. She said:

‘Well, it was put down as depression and anxiety, but I never actually experienced anxiety. And so that was one of the main things, my symptoms were anxiety, and then with the depression side of things, I just kept getting told that my symptoms were stress, or that they were fatigue, or that they were – basically those things that come along with depression were being labelled as what was causing this and then anxiety as well with some of the other symptoms. Yeah, those were the two main things. Even though I never actually experienced anxiety, I was being told I had it because of the physical symptoms I had.’ – Participant 24.

Borderline personality disorder (BPD) is a stigmatised diagnosis that is more frequently applied to women, replacing the historical diagnosis of hysteria (50). One participant expressed their experience of BPD diagnosis as negative:

‘I think with the diagnosis of BPD, they can blame anything and everything on BPD. “Oh, it’s your BPD. So, you are not communicating effectively with people because of your BPD. You’re only suicidal because you cannot manage your emotions, so it must be your BPD,” which is why then the diagnosis of PMDD [premenstrual dysphoric disorder] take ages to come back because, “Oh no, this is just BPD.”’ – Participant 11.

On the one hand, participants in this study appreciated the access to care a diagnosis would give them. On the other, they often resented the way clinicians exercise power against them because of their diagnosis. For example, one participant felt their diagnosis with mental illness disempowered them and created a barrier to being able to make choices and actively participate in their care. They said:

‘I finally found a psychiatrist who is aligned with my values. I’m just waiting on [clinic] to let go of me because they keep extending the [compulsory community treatment order] even though […] I take my own medication, even though if you ask me, even though I’m not at risk, even though the law says, but they still hold on. Anyway, when I see him, I’ll be comfortable to come off the medication under his guidance.’ – Participant 5.

After finding a clinician whose values aligned with her own, this participant was hopeful that she would be able to safely ‘come off the medication.’ This is contrasted with her experience of a clinic that kept extending compulsory treatment.

Many women described how the label of mental illness shaped how clinicians interpreted their behaviour. These interpretations had implications for how their attempts to access physical healthcare were received and responded to. An over-investment in the person’s mental healthcare had implications for their physical healthcare. This participant explained:

‘And the thing is that professionals perceiving what they think they know. Because I had a tooth break and I had to go in for a brain study or a sleep study or something and the dentist had said “look, if you have anything more than milk and a banana, you know, something really, really mild for your teeth, make sure you rinse out with mouth wash.” So of course, whenever I had a cup of tea or a biscuit in hospital for a snack, I’d just go and get a bit of mouth wash. And then suddenly I was obsessive compulsive about using the mouth wash. Even though I tried to explain to them about what the dentist had said, I had it written down on a form somewhere that I was being obsessive compulsive about using mouth wash after eating or drinking. Because that’s apparently one of the, particularly with right front temporal lobe damage, obsessive compulsive disorder is one possible behaviour of concern. Luckily, I missed that one. But to them they were seeing it through their lens of seeing things.’ – Participant 7.

This participant explains how her attempts to follow her dentist’s guidance and look after her teeth are reinterpreted by clinicians as a symptom of mental illness. The experience recounted is an example of diagnostic overshadowing, where the clinicians do not listen to clients because they have already made a judgement about their health needs.

The way that participants were labelled and the impact these labels had on how clinicians and services treated participants undermined the relationships that participants had with health services more broadly. The prescription of medications and how their side effects were dealt with was informed and exacerbated by labels and diagnoses that diminished the knowledge about their health that participants had.

#### Medications and side effects

The women we spoke to felt they had limited control over what medications they were prescribed. They described how their concerns over challenging side effects were minimised or dismissed by clinicians. This was particularly distressing when significant side effects impacted their quality of life and sense of self. One participant said:

‘They [clinicians] do not care because it’s not happening to them. It’s not their life that has to wait three months before you can see the doctor again. It’s not their life that is put on hold. It’s not their life that they have written off.’ – Participant 5.

Side effects from prescribed medications could prevent women from undertaking everyday activities:

‘They [treating team] had me on like four different meds and I was dizzy, and I could not drive, like I do not know how you are supposed to function. Anyway, and they just keep saying “Oh, it’ll settle. Just persevere for six weeks, it’ll settle.”’ – Participant 13.

Another explained how medication for physical health symptoms that are more likely to be experienced by women led to unwanted side effects:

‘I’m also at the moment on a monthly injection to stop my menstrual cycle because of the pain and not wanting to have any more endometrial surgery. That has a lot of side-effects as well and perhaps some weight gain.’ – Participant 15.

This participant went on to explain how they found it difficult to have their concerns over side effects taking seriously:

‘I also had high blood pressure at one point and arrythmia with my heart and blurred vision, yeah, all sorts of things, and the big one that women are socialised not to talk about, too, is like libido or how it affects your sexual relationships, so definitely had impacts on my enjoyment of a sexual relationship.’ – Participant 13.

When concerns were not taken seriously, participants learned from these experiences. One expressed that:

‘I had a case manager, who was a mental health nurse, and she was quite adamant there were no side effects with being on antipsychotics, and the fact that I thought that there were side effects was a delusion, which meant I needed more medication. That was kind of the feedback I kept getting about side effects, so eventually, I kind of gave up getting support for that.’ – Participant 8.

A temporal sense of loss was evident in many women’s accounts:

‘Now it’s taken a long time to come off the wrong drug. That’s years lost to me, or months lost to me. That can never be repaid. That’s a loss that I have to wear because the psych system does not know how to deal with patients in a compassionate manner, in my experience. So, my experience has been very negative of the psych system, I’m really sad to say, because I’m a doctor myself.’ – Participant 5.

The loss of ‘months’ and ‘years’ to side effects contributed to resentment and perception that the ‘psych system’ lacked compassion. One participant expressed that practicing empathy was an overlooked way for clinicians to meet the needs of people who might want support for mental distress:

‘I can say that coming from nursing experience that there is not enough empathy and compassion within the emergency department for people that are not suffering from physical but also mental health issues.’ – Participant 30.

For people prescribed medications for physical and mental health problems, the potential for side effects is compounded.

#### Interactions in physical and mental health

Participants in our study felt that their physical and mental health were linked, but they often had to prioritise one over the other in appointments with clinicians. In many of the previous comments, participants expressed that their mental and physical healthcare needs were often confused, leading to misdiagnosis or medical neglect. One participant explained how their physical and mental healthcare had always been interlinked:

‘It’s been a long journey. So, it started back when I was 14. The biggest thing, I have to say, is that my mental health was what I was initially diagnosed with and that actually affected the care that I got with my physical health. So, my physical health was pushed to the side, and I was told that it was mental health related. It was not until probably 12 months ago to two years ago that I actually started getting diagnosed with the physical things I’ve been experiencing for a long time.’ – Participant 24.

Many of the women we spoke to emphasised that clinicians’ focus on mental health diagnosis contributed to misdiagnosis of physical health concerns and medical neglect. One participant explained how they had to ‘fight’ to have their physical health needs met:

‘So now I’ve got Crohn’s [disease] on top of everything else. And the other thing with Crohn’s is of course, it’s affected by stress, so I can get a flare from stress. I can get a flare from getting a cold or from getting gastro or things like this. So, there is a real direct connection between my physical and mental health with that one. And so that’s been really hard to deal with, because again, generally, the history is, is that I have to fight for them to investigate things.’ – Participant 2.

Another articulated how the interaction of their physical and mental health impacted them with specific reference to the way side effects of medications for treating mental distress caused physical health concerns:

‘Physical and mental health, as your question is, is more that I go up and I burn a lot of energy very quickly, and I lose a lot of weight. So, the physical conditions that I suffer when I’m psychotic is probably quite a healthy condition because I need to lose weight. But some people, not everyone by far, but some people like the psychiatrist, cannot see me. So, there’s conflict. And then, therefore, they medicate, and the medication does the opposite. It makes me heavy. I put on a lot of weight, and I’ve had a lot of problems with weight gain over the 20 years, including knee issues, verging on diabetes, just discomfort, complete sexual dysfunction, absolutely not able to communicate sexually with anyone.’ – Participant 5.

This participant’s experience of side effects was compounded by the indifference of the clinician who prescribed the medication.

One participant expressed their belief that the system was not set up to treat women effectively, and that an overhaul of Western healthcare was needed for women with physical and mental health concerns to have their needs met. They said:

‘Western women have had over 2000 years of putting up with white man’s oppressive, sexualised, abusive, misogynistic, narcissistic behaviour and women are just expected to lie down and take it. So yeah, there’s a lot of compounding factors, it’s not just the physical for me and I want to see that changed.’ – Participant 30.

We can see in this short quotation how, for this woman, resistance and transformation were crucial to ensuring women are able to have their mental health and physical wellbeing needs met. Another indicated that trauma – which we discuss in the following section – compounded their experiences of medical neglect and diagnostic overshadowing:

‘I do think that the public mental health system should still help me, but they do not. It’s all connected, all the complex PTSD medicine, it all intersects with the chronic pain medicine.’ – Participant 9.

The failure of the public mental health system is keenly felt by this participant. For this woman, the distressing side effects of psychotropic medications and ‘pain medicine’ represent the failure of the mental health system to provide effective help and support.

### Outside the doctor’s office

The challenges encountered by participants often intruded into many aspects of their daily lives. Interactions with clinicians shaped how participants experienced the world around them. In turn, participant’s beliefs about what they could expect from clinicians – usually based on experience – shaped the nature, frequency, and timing of their engagement with health services. This had an impact on their lives beyond direct interactions with clinicians.

#### Trauma

Trauma was a prominent theme in the participants’ accounts. Trauma was both something participants directly sought help for, and something that was in the background when the person was seeking help for other health concerns.

Many women described how previous experiences of trauma influenced their experience of certain health procedures. For example:

‘I think the other unfair expectation is I think that medical staff expect you to be able to cope at a level they would be able to cope. So, I think that that happens a lot too. They probably look at you and think, “Well, this is not a big deal. I can do this. This is just a blood test,” or this is just a whatever procedure. It is not realising for example or, “This is just a pap smear.” But not realising well I’ve been someone that’s endured a sexual assault, so that’s not just a pap smear for me.’ – Participant 2.

It is important to understand that trauma shaped how people accessed and experienced all forms of healthcare. For one participant, engagement in the mental health system was directly caused by her ongoing experience of trauma. She said:

‘My inpatient stays are triggered by my trauma surfacing’ – Participant 17.

Another took issue with the label of mental illness because they felt that their distress was a response to not being taken seriously – not a symptom of illness. They said:

‘I’m not mentally sick, I’m somebody that suffers from complex post-traumatic stress disorder because I’ve been put in the too-hard basket and I’m not going to allow people to do that to me anymore and why should I?’ – Participant 30.

This quotation highlights how women often felt they had to assert their right to have their physical and mental health needs addressed. In a similar vein, another participant reflected that clinicians should approach care in a more trauma-informed way, by offering women an option for the gender of their clinician. She said:

‘They should ask if you are okay, if… “Do you have a problem seeing a male dentist?” or “Would you like a lady to do your pap smear?” because I have a lady doctor that does my pap smears. I will not go to a man for that stuff.’ – Participant 29.

Mental health treatments could in and of themselves be experienced as distressing and traumatic, particularly for women who had experience coercion within therapeutic interventions. This often contributed to distress:

‘I still have the effects of the forced… the needles… so I still have those memories come back sometimes at night, that’s part of why I struggle to sleep at night sometimes because the care at night was a lot worse.’ – Participant 13.

We can see here how mental health services can contribute to trauma and shape future interactions with mental health services.

#### Financial concerns

Finances were a significant barrier to accessing appropriate physical and mental healthcare. In Australia, where this study was conducted, there is a two-track system where some medical services are fully or partially funded by the government. When an insufficient number of clinicians work for the amount funded by the government, clients are obligated to pay out of pocket. Clients can also purchase private health insurance which assists in funding some services.

For participants in this study, money was understood as having power to alleviate suffering through the purchase of medical services and/or medications. One participant said:

‘Why should women be seen as an excuse because they go through menopause? I can tell you now, not all women have the luxury to spend the money to go to these fancy hormonal clinics to get the best treatment that they possibly can. Let us look at the women that are on the socioeconomic disadvantage, that cannot afford to go to clinics, that cannot afford HRT [hormone replacement therapy] or are afraid of HRT because it’s going to give them cancer or cause a clot in their brain.’ – Participant 30.

People with disabilities in Australia may be eligible for the Disability Support Pension – a fortnightly allowance administered by the federal government via the social service agency, formerly known and colloquially referred to as Centrelink. However, participants struggled to access this payment. One said:

‘And even Centrelink; I just had to go through, again, a review with them, to say … even though I was on the Disability Support Pension, a couple of weeks ago, I had to prove again to them that I have permanent disabilities, and my GP and I thought, “Okay, it’s a good opportunity for me to try and look for work,” and she said, “Let us make it a minimum of eight hours,” because I know that that is the requirement.’ – Participant 11.

One participant expressed that the financial cost of managing their physical and mental healthcare had lowered the quality of their life by introducing financial strain. They said:

‘Every penny I have ever earnt or had; I’ve spent my whole life trying to get better which has meant that I have not been able to go on holidays. I have not been able to buy a house or get a new car or a lot of things that other people are able to do when they have fully functioning lives. Yeah, there was a big financial toll because it costs so much money to try to get better and to maintain the level of health that you have even got. Yeah. And it requires more money than the disability pension can give you.’ – Participant 2.

Meeting the prescribing directions of clinicians was a notable hardship for some. One participant said their employer:

‘I needed medication. Now I take many medications. Every month I pay $50 for medication now.’ – Participant 27.

Financial stress meant that participants had to prioritise their health over other things in their lives to justify the high cost for healthcare. However, for clinicians, seeing and billing patients is the bread and butter of their profession. This may have contributed to participants not getting what they need from consultations – a theme we pick up on in the discussion.

#### Reliance on internal resources

Limitations within the service systems and discriminatory responses encountered when attempting to access support meant that participants developed internal resources and took an active role in their health journeys. One participant highlighted the inequity and injustice that eroded women’s sense of self:

‘We are real people. We are real people that are struggling and if we cannot navigate our own pathways to find our own services for us, nobody is going to do it for us.’ – Participant 30.

This quotation highlights the isolation and need for self-reliance that participants developed. Faced with barriers to appropriate and supportive healthcare, they often had to take charge and rely on themselves to manage their health. One participant said:

‘I can say that having to juggle family life, disability, physical health, and mental health, it’s not been easy. It’s been a massive challenge, and I do not expect a lot of people to understand that. The only time that they do understand it is when it actually happens to them and then all of a sudden there is this shift in empathy like, “Oh, we now get what that person is saying.” So yeah, I do agree that mental health and physical health, they go hand in hand.’ – Participant 30.

Women often found they had to stand up and challenge inappropriate and stigmatising responses from health and social care practitioners. This was particularly the case when managing unhelpful or inaccurate advice from clinicians. One participant said:

‘You’re going to be coming against people who will discriminate you, who do not have an understanding of what your conditions are like, who will not get what mental health is like, who will be in the dark, who will say you are making things up and you have just got to know that this is… people are going to respond to you in very different ways, but you need to be determined to get through and to realise that you are the best judge of your health conditions and how they should be managing, how you are the one who knows how you should manage each condition. The doctors can provide you advice, and the allied health, but they may not be correct.’ – Participant 11.

Among these women, there was an acceptance that when using services, they will encounter ‘people who will discriminate’ and ‘who do not have an understanding’ within the health system, creating a need for them to self-advocate in order to meet their needs. Cultivating internal resources were essential to combatting inadequate and stigmatising responses to their health needs.

Participants in our study had multiple responsibilities in their daily lives, including work, study, and children, which they managed in addition to their physical and mental health. One participant identified this as a disabling factor of women’s lives, linking their experience with a broader movement for women’s equality, highlighting and resisting the conditions that lead to women being designated as “hysterical.” They said:

‘Why do women always get seen as psychotic or hysterical?’ – Participant 30.

## Discussion

Women in this study experienced a variety of barriers to accessing healthcare including discrimination related to their gender, mental health status, and physical health status. These women are a part of a legacy of the over-pathologisation of women’s health needs, particularly mental health needs (41). This paper fills a gap in the literature on the experiences of people diagnosed with mental illness and physical health concerns by focussing only on the experiences of women.

The women in this study expressed a variety of challenges in having their physical and mental health needs met. Some of these challenges may also be experienced by people of other genders, however, women had their own unique experiences of each barrier. For example, it is well documented that the prescription of antipsychotics can lead to weight gain across all genders (51, 52). However, weight gain may be something women are more likely to experience as distressing and experience weight discrimination over (53, 54). As illustrated above, women in this study experienced weight gain as distressing. Similarly, women may obscure their mental health symptoms because of the likelihood of discrimination once diagnosed (55).

Throughout all identified themes in our study, women expressed a need to be taken seriously by clinicians. Some felt that their physical health symptoms were explained away as mental health symptoms – women are more likely to be diagnosed with mental illness (56), and so may be more vulnerable to clinicians falsely attributing their physical health concerns to mental illness. Women with mental illness are more likely to be diagnosed with breast cancer at later stages of the illness, possibly because early symptoms are misrecognised as mental illness (57). A study by Behzad Karami et al. (58) found that women with disabilities – who are frequently engaged with health services – would benefit from clinicians taking their knowledge and preferences seriously when making treatment decisions. The recommendation these authors make from this is that clinicians should have training for how to work with women with disabilities. Another study by Hayman et al. (59) made a similar recommendation that clinicians would be better positioned to meet women’s physical health care needs if they also assessed mental health symptoms, particularly stress. The findings in our study suggest that clinicians to need to be more attentive to the needs of the women they work with, however, identifying the problem as a lack of training serves to cultivate further distance between clinicians and clients. Rather, the women in our study did not want clinicians to undertake short courses in how to talk to them about their needs – they wanted clinicians to listen to them.

Further research should consider the experiences of women who are further minoritized, for example, through racialisation. Women from non-white cultural backgrounds in the UK were less likely to get the healthcare they needed (60). Indigenous women endure specific barriers including access to ‘private healthcare, long waiting times associated with the public health system, lack of culturally competent healthcare practitioners, high health staff turnover as well as systems failures concerning diagnosis, treatment and the provision of effective follow-up care planning’ (61). Indigenous women with disability face further disablement through the lower likelihood of being able to find and access a disability service that meets all of their healthcare needs and is sensitive to their cultural identity (62). Like participants in this study, prior negative experiences with health services also reduces their engagement with future clinical services, further compounding and creating a cycle of chronic inequality (61). There is a need for further research into the experiences of women from culturally and linguistically diverse (CALD) backgrounds, who are less likely to have their healthcare needs met (60), particularly in low-income countries (63).

### Limitations

There is an opportunity to address the experiences of transgender women and people assigned female at birth but who are not women. While we did aim for variation in recruitment, no transgender or gender diverse participants enrolled in our study, which is a limitation. These voices are underrepresented in this literature, and people who do not identify as cisgender are more likely to experience mental illness (64, 65), in part due to the impacts of marginalisation, discrimination, minority stress, poverty, socio-economic inequity, healthcare inequity, and exposure to violence (66–70), and subsequently, greater vulnerability to physical health concerns (71, 72).

## Conclusion

Women diagnosed with mental illness and physical health concerns deserve access to the medical, social, economic, spiritual, and psychological care. This study has illustrated that for the women we spoke to, all these types of care intersected. All the women in this study had demonstrated they regularly evaluated their own needs and had strategies to have these met. However, too often the options available in the clinical spaces and the behaviour of clinicians served as a barrier both to women getting the healthcare they needed and to having their wishes about treatment met.

## Data availability statement

The datasets presented in this article are not readily available because of the risk of reidentifying participants. Requests to access the datasets should be directed to T.Zirnsak@latrobe.edu.au.

## Ethics statement

The studies involving humans were approved by Royal Melbourne Institute of Technology Human Research Ethics Committee (Project Number (#23687)). The studies were conducted in accordance with the local legislation and institutional requirements. The participants provided their written informed consent to participate in this study. Written informed consent was obtained from the individual(s) for the publication of any potentially identifiable images or data included in this article.

## Author contributions

TZ: Data curation, Formal analysis, Investigation, Methodology, Writing – original draft. RE: Conceptualization, Data curation, Formal analysis, Investigation, Methodology, Writing – review & editing. GM: Conceptualization, Formal analysis, Investigation, Methodology, Writing – review & editing. EC: Formal analysis, Investigation, Methodology, Project administration, Writing – review & editing. CG: Conceptualization, Investigation, Methodology, Project administration, Writing – review & editing. NH: Conceptualization, Data curation, Formal analysis, Investigation, Supervision, Writing – review & editing. RR: Data curation, Funding acquisition, Investigation, Methodology, Project administration, Resources, Supervision, Writing – review & editing. CM: Conceptualization, Data curation, Funding acquisition, Investigation, Methodology, Project administration, Resources, Supervision, Writing – review & editing.
