# Supplementary material for: “I have to fight for them to investigate things”: a qualitative exploration of physical and mental healthcare for women diagnosed with mental illness
Source: Front Public Health. 2024 Apr 30;12:1360561. doi: 10.3389/fpubh.2024.1360561 (PMC11095107; doi:10.3389/fpubh.2024.1360561)
Supplement: Supplementary file 1 [file Data_Sheet_1.pdf]

Supplementary File: Interview Schedule

Equally Well Healthtalk: Physical Health of People with Lived Experience of Mental Illness: Interview Topic Guide

Preamble

In this interview, you are invited to talk about your experiences of your physical and mental health. We are interested in hearing about these experiences in the context of your overall life. You can mention as much as you feel is relevant and important to you.

In the first part of the interview I will ask you to tell me about your experiences. During this stage I may ask you a few follow-up questions to clarify things you have told me or ask for a little more information. Once you have finished your story, I may have a few extra questions about other aspects of your experience we are interested in. If at any point you wish to stop, or if there are questions you don't wish to answer, just let me know.

Part One: Invitation for Participant to Tell Their Story

Could you tell me about your experience your experiences of your physical health and the care and treatment you have accessed since your first experience of a mental health issue? Please talk about anything that you feel is important and you are free to share as much as you are comfortable with.

Leave participant to talk as long as they want.

Part Two: Follow Up

(*Once the person has finished telling their story, follow up on the aspects below if they have not already been touched on. This will vary between interviewees depending on how expansive / detailed their initial narrative is – some people say very little while others give lengthy accounts.)*

1. General health

- Main concerns (physical & mental health), impacts on life over time and on a day-to-day basis
- Healthcare services accessed (all kinds)

2. Mental health

- First experiences of mental health problems (symptoms, seeking help, diagnosis)
- Mental health medications / treatments – information, choice, experience, unwanted effects

- Care for mental health accessed
  - type of care (e.g. medical care, allied health care, peer support, informal support (e.g. family & friends), self-care)
  - setting (e.g. community, hospital)
  - involuntary vs voluntary treatment
  - accessibility (cost, location, other barriers)
  - experiences of health professionals re: mental health
  - what helps, what doesn't
- Current concerns about mental health / approach to managing these

3. Physical health

- First experiences of key physical health problems (symptoms, seeking help, diagnosis)
- Physical health medications / treatments – information, choice, experience, unwanted effects
- Care for physical health accessed
  - type of care (e.g. medical care, allied health care, peer support, informal support (e.g. family & friends), self-care)
  - setting (e.g. community, hospital)
  - accessibility (cost, location, other barriers)
  - experiences of health professionals re: physical health
  - what helps, what doesn't
- Current concerns about physical health / approach to managing these

4. Interrelationship between mental and physical health

- Impact of mental health issues on physical health (e.g. symptoms, treatments)
- Impact of physical health on mental health
- Management of / approach to physical health by mental health professionals
- Management of / approach to mental health by 'physical' health professionals
- Coordination / continuity of care

5. Personal and social impacts / dimensions of mental and physical health concerns

- Relationships (family, friends, partner)
- Work or study (including volunteering)
- Financial / economic wellbeing
- Identity / sense of self

6. Reflections and interview close

- Thoughts about the future regarding own health
- What optimal mental and physical health care would look like (for participant)
- Views about what could be improved in relation to the physical health care of people with lived experience of mental illness in terms of:

    - Health services
    - Health practitioners
    - Peer support services
    - Other ideas (e.g. other institutions such as education, workplaces etc, social values / attitudes)
- Advice / suggestions for others:
    - people with lived experience of mental illness confronting physical health concerns
    - healthcare practitioners
    - policymakers
- Anything else participant would like to add

Thank you for your time.
